# How Inflammation Impinges on NAFLD: A Role for Kupffer Cells

**DOI:** 10.1155/2015/984578

**Published:** 2015-05-18

**Authors:** Nádia Duarte, Inês C. Coelho, Rita S. Patarrão, Joana I. Almeida, Carlos Penha-Gonçalves, M. Paula Macedo

**Affiliations:** ^1^CEDOC, NOVA Medical School, Faculdade de Ciências Médicas (NMS/FCM), Universidade Nova de Lisboa, Edifício CEDOC II, Rua Câmara Pestana, Nos. 6, 6A, 6B, Laboratory 3.8, Piso 3, 1150-082 Lisboa, Portugal; ^2^Instituto Gulbenkian de Ciência (IGC), Rua da Quinta Grande 6, 2780-156 Oeiras, Portugal; ^3^APDP-ERC Portuguese Diabetes Association Education and Research Center, Rua do Salitre, No. 118-120, 1250-203 Lisbon, Portugal

## Abstract

Nonalcoholic fatty liver disease (NAFLD) is rapidly becoming the most prevalent cause of liver disease worldwide and afflicts adults and children as currently associated with obesity and insulin resistance. Even though lately some advances have been made to elucidate the mechanism and causes of the disease much remains unknown about NAFLD. The aim of this paper is to discuss the present knowledge regarding the pathogenesis of the disease aiming at the initial steps of NAFLD development, when inflammation impinges on fat liver deposition. At this stage, the Kupffer cells attain a prominent role. This knowledge becomes subsequently relevant for the development of future diagnostic, prevention, and therapeutic options for the management of NAFLD.

## 1. Introduction

We are facing a rampant epidemic of nonalcoholic fatty liver disease (NAFLD) which afflicts adults and children and is frequently associated with obesity and insulin resistance. In Europe and in the United States, it is estimated that 3 out of 10 adults developed NAFLD [1, 2]. As NAFLD is mostly asymptomatic, such estimates have a high degree of uncertainty. For example, European data suggest that NAFLD prevalence can fall in a wide range, between 2 and 44% in the general population, and is present in 42.6–69.5% of people with type 2 diabetes [3]. NAFLD prevalence in obese children was found to reach values as high as 36–44% regardless of the diagnostic criteria. A study that evaluate children in the Japanese population observed that fatty liver may occur in children as young as 6 years of age and is directly related to the degree of obesity and specifically to the abdominal subcutaneous fat thickness [4]. Remarkably, women with history of gestational diabetes have a greater prevalence of NAFLD and prospective studies estimate that these women are at a higher risk of developing diabetes [5]. Another group that presents high prevalence of fatty liver is the elderly representing a public health concern for developed countries with aged populations. The Rotterdam study describes that NAFLD was present in more than one-third of the assessed elderly with a sturdy association with dysglycemia, dyslipidemia, and abdominal fat [6]. More interestingly, the subjects with advanced age show a decrease incidence of NAFLD suggesting the possibility of positive selection of elderly without NAFLD [6].

The estimation of NAFLD impact at population level poses two questions. (1) Realistically, should we assess NAFLD recurrently in the population? (2) Is there a good, easy, and not costly way of doing it? At a first glance, NAFLD could be seen as a response mechanism to deal with increased amounts of lipids intake, representing a protective mechanism. To identify the disease in early stages, we would need liver biopsies, the recognized gold standard for NAFLD diagnosis. Obviously, the invasive nature of such procedure excludes its widespread use. The more widely used ultrasound, however, will not inform us of the grade of liver inflammation. Magnetic resonance imaging and computed tomography even though more sensitive are expensive and cannot distinguish between simple steatosis and the severe form of NAFLD termed nonalcoholic steatohepatitis (NASH) [7]. Alternatively, several indexes composed of physical and biochemical parameters such as the fatty liver index (FLI) have been proposed to evaluate NAFLD at the population level [8]. Undesirably, such methods do not detail the degree of fat neither the grade of inflammation in the liver. In this scenario, NAFLD evaluation calls for novel biomarkers that reflect relevant NAFLD physiopathology mechanisms providing a solid basis for diagnosis and possible innovative therapeutic approaches.

## 2. Ectopic Accumulation of Triglycerides, Hepatic Steatosis

Triglycerides (TG) accumulation is an efficient energy storage mechanism. When compared to carbohydrates (4,5 kcal/g) or proteins (4 kcal/g), TG provides higher caloric intake (9 kcal/g) [9]. As so, it is advantageous for the organism to convert carbohydrates and amino acids into TG to be stored in adipose tissue, in order to be used in times of fasting or prolonged exercise [10].

Physiologically, TG are a way of balanced energy storage. However, over the last decades, the excessive consumption of food rich in fat and sugars, associated with excessive caloric intake, has led to alterations in lipids and glucose metabolism. The current recommendations for adults are that 20% to 35% of the daily calories ingested are derived from dietary lipids. Nevertheless, this value is estimated to be around 40% in western diets [11]. The imbalance in lipids metabolism is tightly associated with several diseases such as obesity, diabetes, and NAFLD [12], representing an emerging health concern.

Overconsumption of fat and sugar ends up in ectopic lipids accumulation, with the liver being one of the prime targets in this process. Hepatic accumulation results from a disparity between lipids availability (from circulating lipid uptake and/or* de novo* lipogenesis (DNL)) and lipid disposal (via fatty acid oxidation and/or production VLDL) in the liver [10, 13, 14]. This lipid deposition within hepatocytes is described as hepatic steatosis and it has been suggested to occur due to three main factors: (1) TG coming from lipolysis of adipose tissue; (2) TG synthetized from DNL; and (3) TG from diet; with each one contributing about 59%, 26%, and 15%, respectively, to TG accumulation [15]. The overflow of free fatty acids (FFAs) coming from lipolysis of adipose tissue is the main contributor to hepatic steatosis [15, 16]. Exacerbated accumulation of fat in the adipose tissue culminates in adipocyte hypertrophy with limitations to lipid storage and recruitment and activation of inflammatory mediators [10, 14].

Hepatic steatosis occurs primarily by the formation of lipid droplets [17] considered to be a noncytotoxic structure of TG storage [10]. Lipid droplets of TG have a protective effect against lipids deposition, preventing the formation of toxic lipids intermediates [18]. Conversely, FFA coming from circulation,* per se*, may be toxic to hepatocytes, since they are able to stimulate innate immune system receptors, such as toll-like receptors (TLRs) [19], contributing to the inflammatory response. In addition, FFA may also impact differently on hepatocytes, depending on their nature and not specifically on their quantity. Other studies have shown that the ratio of monounsaturated fatty acids (MUFA) to saturated fatty acids (SFA) determines whether liver cells are damaged by FFA flux [20].* In vitro* experiments also demonstrated that incubation with palmitate (SFA) lead to cell toxicity, with ROS production and cell death, contrary to incubation with oleate (MUFA). Oleate alone increased TG accumulation without cell damage and in combination with palmitate and promoted lipid droplet formation abolishing the palmitate effect [21]. Another study showed that increased levels of stearoyl-CoA desaturase-1 (SCD1), the enzyme that catalyzes the desaturation of SFA palmitate to the MUFA oleate, protects from cell death in the context of FFA overflow [20]. Similar results have been observed in animal models of progressive liver disease, where SCD1 expression was highly suppressed with consequent decrease in esterification of SFA into TG, thus promoting liver injury [20, 22].

The exact mechanisms by which SFA-induced lipotoxicity occur are not well established. It is known that these FFA promote the release of several proinflammatory cytokines and trigger endoplasmic reticulum (ER) and oxidative stress [23]. ER is responsible for an adequate protein synthesis, folding, and secretion and its disruption may compromise cell viability. Excessive SFA entering hepatocytes are conducted to the formation of saturated phospholipids that are then integrated into ER membrane bilayers, affecting ER membrane fluidity [24]. Therefore, the process responsible for degrading misfolded proteins, the unfolded protein response (UPR), is activated, culminating in apoptosis when the number of unfolded proteins cannot be processed [25].

Increased oxidative stress was observed in NASH patients and in animal model in association with accumulation of oxidized lipids [26]. Taken together, evidences show that SFA have a huge impact on hepatocytes viability. High fat and high carbohydrate diets have a vast SFA component, with higher SFA/MUFA ratio, which is critical to NAFLD progression.

Highlighting the role of diet composition, it was shown that NAFLD and NASH patients had a dietary intake richer in SFA and cholesterol and poorer in polyunsaturated FFA and fibers [27]. A lower intake of proteins and zinc was also shown in these patients [28].

## 3. The Role of Carbohydrates

Evidence suggests that high sugar intake combined with lipid oversupply exacerbates liver pathology. Fructose is a popular monosaccharide used industrially in the form of high-fructose corn syrup (45% glucose; 55% fructose) to sweeten foods and beverages [29]. Consumption of fructose has markedly increased and is estimated to be 2-3 times greater in patients with NAFLD, compared with patients with other liver diseases or healthy control individuals [30, 31].

In humans, all fructose that is ingested is metabolized by the liver [32]. In contrast to hepatic glucose, fructose is neither converted into glycogen nor stimulates postprandial ghrelin or insulin secretion, failing to initiate the central satiety response [33]. Moreover, this carbohydrate was reported to stimulate hepatic DNL through increasing the expression of two transcription factors, sterol regulatory element-binding protein-1c (SREBP-1c) and carbohydrate-responsive element-binding protein (ChREBP), that regulate the activity of lipogenic enzymes [34]. Comparison of fructose and glucose intake in human studies revealed that the first is more lipogenic than the later (10% versus 2% increase in DNL) [35]. A recent study suggested that fructose consumption may specifically promote lipid deposition in visceral adipose tissue, particularly in men, whereas glucose consumption appears to favor subcutaneous adiposity [36].

Fructose* per se* plays a role in triggering inflammation, possibly through the disruption in gut microbiota and increased mucosal permeability. Spruss et al. [37] showed that mice exposed to fructose exhibited markedly intestinal bacterial overgrowth and increased intestinal permeability, leading to an endotoxin-dependent activation of hepatic Kupffer cells (KCs). Increased plasma concentration of tumor necrosis factor (TNF)-*α*, portal lipopolysaccharide (LPS), and myeloid differentiation primary response gene (MyD)88 have observed these mice [37].

Taken together, these results show fructose as an important dietary contributor to NAFLD pathogenesis and severity [38], being more detrimental than other carbohydrates.

## 4. Nonalcoholic Steatohepatitis and the Multiple Hit Hypothesis

The severe form of NAFLD disease is termed as NASH and is characterized by the presence of hepatocellular injury, lobular and/or portal inflammation, and frequently, deposition of collagen fibers [39]. Although patients with steatosis are at increased risk of progressing to severe forms of liver disease, NASH may develop in absence or in presence of very little steatosis [39]. This indicates that steatosis and inflammation may not always be sequential events. The initial view that considered steatosis as a primary hit and gut-derived endotoxin as the second hit determining progression of the disease is now followed by a more complex model where many hits may act in parallel [40]. Dysregulation in lipid metabolism associated with diet-induced changes in microbiota and increased proinflammatory signaling to the liver together with the liver local inflammatory response is amongst the hits involved in NASH [40]. In light of this model, hepatic inflammation may be either the cause or consequence of steatosis, but it is always determinant in tipping the balance towards NASH and worse prognosis.

## 5. The Gut/Adipose Tissue/Liver Axis

Recent evidences place the liver and its local response to metabolic and immune mediated disturbances in the gut-adipose tissue axis [41]. Understanding how this interplay is disturbed in NASH constitutes the current challenge. Certain dietary factors may promote lipotoxicity and/or induce alterations in the gut microbiota that may result in abnormal activation of the gut immune response and bacterial product translocation with systemic impact on immune system modulation [42]. It was observed that mice deficient in inflammasomes, which are cytoplasmic multiprotein complexes that sense endogenous or exogenous pathogen-associated molecular patterns (PAMPs) or damage-associated molecular patterns (DAMPs), showed perturbations in intestinal microbiota. When placed under methionine-choline-deficient (MCD) diet for 4 weeks, known to cause similar features of human NASH, the inflammasome deficient mice developed severe symptoms like a marked impairment in glucose homeostasis, increased weight gain, and NASH development that could be transferred to wild-type cohoused mice. These experiments clearly associate changes in microbiota with NAFLD development and obesity [41]. In support, high levels of endotoxin in circulation have been associated with NAFLD development in both human and mouse studies [43]. Disturbances in nutrient absorption are other suggested potential adverse effects of gut microbiota imbalances [40, 44]. Complex carbohydrates can be fermented by certain gut bacteria and converted into short-chain fatty acids that stimulate DNL [43]. Adipose tissue dysfunction in its turn is highly linked to the development of hepatic steatosis. Excessive adiposity leads to insulin resistance, augmented lipolysis, increase FFA circulation, and secretion of inflammatory mediators that promote liver steatosis and organ inflammation [10, 45].

## 6. Main Inflammatory Mediators

### 6.1. The Innate Immune Receptors

The innate immune system recognizes immunogenic signals through pattern recognition receptors (PRRs). Bacterial products such as LPS, lipoproteins, flagellins, and peptidoglycans are amongst the PAMPs recognized by these receptors. Endogenous DAMPs like heat shock proteins, high mobility group box 1 (HMGB1), and breakdown products of extracellular matrix liberated from tissue damage and cell death also signal through PRRs [46]. PRRs have been implicated in the pathophysiology underlying NASH. TLRs and in particular TLR4 that recognizes LPS from gram-negative bacteria and TLR9 that recognizes bacteria-derived CpG-containing DNA have been proven critical for several aspects of disease [47, 48]. Nucleotide oligomerization domain- (NOD-) like receptors (NLRs) are intracellular PRRs that are part of the inflammasomes briefly mentioned above. Inflammasomes are multiprotein complexes that through NLRs sense intracellular danger signals and initiate an activation cascade of events that culminate with autoactivation of caspase 1 and cleavage of prointerleukin- (IL-) 1*β* and proIL-18 into mature forms [42]. By controlling the release of these important inflammatory cytokines, Inflammasomes play an important role in the inflammatory process underlying NASH.

TLR signaling and inflammatory cytokines like IL-1 regulate the activation of the transcription factor NF-*κ*B, a critical modulator of liver immune responses [49].

### 6.2. TLR4, TLR9, and Inflammasomes

TLRs are found in several cellular components like plasma membrane and endosomes and the majority associates with a common adaptor molecule, MyD88 through Toll/IL-1 receptor (TIR) domain. Trafficking and location of TLRs within the cell are important for ligand binding and downstream signaling transduction. TLR4 is located mainly in the plasma membrane and associates with the LPS binding protein CD14 and myeloid differentiation- (MD)-2 molecule to recognize LPS. TLR4 can also bind to TIR-domain containing adapter inducing interferon-*β* (TRIF) to induce type 1 interferon (IFN) through Myd88 independent pathway [50]. Endosomal TLR4 seems to recruit preferentially TRIF while plasma membrane TLR4 recruits MyD88 and engages MAP kinases and NF-*κ*B signaling [51]. Amelioration of steatosis and NASH achieved by TLR4 deficiency in several diet-induced mouse models of NAFLD has placed this molecule at the center of inflammation driven pathology [37, 43, 48, 52, 53]. Expression of proinflammatory cytokines is suppressed in TLR4 knockout mice under NAFLD prone conditioning [43]. Increased LPS translocation into portal vein is thought to activate the liver resident macrophages, the KCs, inducing the production of inflammatory cytokines and type 1 IFN, contributing to leukocyte infiltration and fibrogenesis [50]. Markedly, wild-type mice on chow diet when infused continuously with low-dose LPS developed hepatic steatosis, insulin resistance, and weight gain [54]. TLR4, while mostly expressed on KCs, is also found on other liver cells like hepatocytes and hepatic stellate cells (HSCs), the main producers of extracellular matrix in the fibrotic liver. Interestingly, TLR4 expression on HSC was critical to sensitize these cells to profibrogenic tumor growth factor (TGF*β*) signaling in a Myd88 dependent path in a model of hepatic fibrosis [52]. TLR4 was also important for HSCs mediated recruitment of KCs. TGF*β* secretion by KCs is required for fibrosis development. However, absence of TLR4 on KCs did not affect the fibrogenic process in this model [52].

TLR9 is located in the endosomal compartment and binds to an unmethylated CpG motif that is very prevalent in bacterial DNA. TLR9 knockout mice under a choline-deficient amino acid defined (CDAA) diet for 22 weeks, known to induce NASH with characteristic steatosis, inflammation, and fibrosis, showed amelioration of disease through suppression of IL-1*β* secretion by KCs [55]. These results link TLR9 signalling to inflammasome activation.

The most studied inflammasome is NLRP3 (also designated by NALP3 and cryporin) that comprises the NOD-like receptor NLRP3, the apoptosis-associated speck-like protein containing a caspase recruitment domain (Asc), and the effector molecule procaspase 1. Inflammasome activation is regarded as a two-step process in which the first signal, frequently TLR signaling, upregulates inflammasome expression and the second signal triggered by an inflammasome ligand results in activation [42]. LPS, DNA, SFA, amyloid, cholesterol, cathepsin *β*, and reactive oxygen species (ROS) among others have been suggested as NLPR3 activators. Danger signals released from fatty acid-treated hepatocytes were also shown to induce inflammasome activation in liver mononuclear cells [42, 47]. Asc knockout mice showed exacerbated MCD-induced NASH driven by abnormal gut microbiota and increased translocation of TLR ligands into the portal vein [41]. On the other hand, high fat feeding in mice lacking Asc resulted in diminished weigh gain, fat mass, and improved insulin resistance [56, 57], discrepancies that could be attributed to heterogeneities in intestinal microbiota [41]. Another study showed increased NLPR3 and caspase 1 expression in the liver of NASH patients [58]. Augmented levels of these molecules in adipose tissue are also correlated with type 2 diabetes in obese patients [59]. It is important to note that inflammasome activation might lead to different outcomes according to the tissue or cell-type that receives the stimuli [42].

### 6.3. IL-1, TNF*α*, and IL-6

High fat diets in mice lead to increased hepatic expression of NF-*κ*B, hepatic steatosis, and rise in IL-6, IL-1*β*, and TNF*α* gene expression [39].

Supporting a role for inflammasome in NASH, IL-1 receptor (IL-1R), IL-1*β*, and IL-1*α* knockout mice showed attenuation of liver pathology induced by high fat diets [42]. Activation of IL-1R by IL-1 resulted in the activation of the transcription factor NF-*κ*B [49]. IL-1 forms an autoregulatory loop as it induces both the expression of its own precursors and inflammasome components, suggesting that small increases in this cytokine might have significant biological effects [42].

TNF*α* overexpression is viewed as the hallmark of inflammation in obesity and NAFLD pathology and a major link to insulin resistance. Upon binding to its receptor, TNF*α* can activate proapoptotic or antiapoptotic signaling cascades leading to NF-*κ*B activation, thus regulating cell viability, inflammation, metabolism, and other cytokine productions [39]. It is overproduced in adipose and muscle tissues of obese humans and in rodent models of obesity. TNF*α* or TNF*α* receptor knockout obese mice have improved insulin sensitivity compared to wild-type controls [60]. TNF*α*, through NF-*κ*B both promotes and is activated by insulin resistance and is involved in liver inflammatory and metabolic alterations [39]. Additionally, TNF*α* antagonizes the anti-inflammatory cytokine adiponectin. Adiponectin is produced by adipocytes and sensitizes cells to insulin. It also promotes fatty acid oxidation with marked anti-inflammatory and antilipogenic effects in the liver [39]. Increased levels of circulating TNF*α*, on the other hand, correlate with NAFLD disease activity as measured by histological parameters in NAFLD patients. Likewise, TNF*α* and TNF*α* receptor gene expression was increased in hepatic and adipose tissues in NASH patients [50]. Experimental models of insulin resistance demonstrated amelioration of inflammation, increased insulin sensitivity, and improved steatosis upon treatment with infliximab, a potent TNF*α* neutralizing monoclonal antibody. However, highlighting the complexity of inflammatory signaling, infliximab treatment did not improve insulin sensitivity in human obese insulin resistant individuals. Furthermore, pentoxifylline, a phosphodiesterase inhibitor, prevented TNF*α* production with only modest amelioration of insulin resistance in small studies of NASH patients [50].

Visceral adipose tissue of obese individuals secretes increased amounts of IL-6 when compared to subcutaneous fat. Increased levels of plasma IL-6 were associated with augmented inflammation and fibrosis in NAFLD patients [39]. Adipocyte-derived IL-6 was shown to regulate hepatic insulin resistance via upregulation of suppressor of cytokine signaling 3 (SOCS3) which in turn induces an increase in SREBP-1c and DNL [50].

Interestingly, IL-6 and TNF*α* deficient mice displayed reduced hepatocarcinogenesis after high fat diet and upon carcinogenesis induction by diethylnitrosamine (DEN) treatment [61]. On the other hand, IL-6 deficiency was associated with increased hepatocyte injury and apoptosis in a mouse model of liver fibrosis [62]. In this model, IL-6 production by nonparenchymal cells was protective possibly by downregulating HSC activation [62].

## 7. Kupffer Cells

The liver is structurally and functionally heterogeneous. Parenchymal cells, that is, hepatocytes, are the most numerous and comprise 60% of the total liver cells and 80% of the volume of liver. Nonparenchymal cells (NPCs) represent around 20% of the liver cells and include sinusoidal endothelial cells (20% of liver cells), KCs (approximately 15% of liver cells), and hepatic stellate cells (5–8% of liver cells). Other immune cell populations mostly of natural killer T cells (NKTs) comprise a minor fraction of NPCs [63]. Hepatocytes perform the majority of liver functions. Nevertheless, evidence shows that both, under normal and pathological conditions, substances released from neighboring NPCs, mainly KCs, regulate several hepatocyte functions [64].

KCs are the resident macrophages of the liver comprising the largest tissue specific population of macrophages in the body. The major immune function of KCs in healthy liver is to phagocyte and present pathogens entering from portal vein and arterial circulation, constituting one of the first lines of defense of the organism. Under these circumstances, they present mostly a “tolerogenic” phenotype preventing undesirable immune responses to the common gut-derived antigens they are constantly in contact with [65].

KCs are differently distributed in the liver and it is possible to find two different populations regarding its localization. Large KCs are localized in the periportal zone and have increased phagocytosis and increased production of biological mediators. These large KCs can be identified by the expression of CD163, a scavenger receptor. KCs can be also identified by the general macrophage marker F4/80 or by CD68, which is present in all KCs regardless of their location [65]. The KC population can be replenished by bone marrow-derived monocytes [66]. Whether these recruited cells form a fully independent population when compared to liver self-renewed macrophages of fetal origin is still debatable. In addition, macrophages present a wide range of phenotypic and functional plasticity depending on the environmental stimuli they perceive [67]. Classical activation of macrophages with IFN-*γ* and LPS induce differentiation into the M1 phenotype marked by the release of proinflammatory cytokines like TNF-*α*, IL-1, and IL-12. Alternative activation into M2 phenotype is more heterogeneous as different stimuli (such as IL-4 or IL-10) can induce different phenotypes. Typically, increased expression of arginase 1, secretion of immune-modulatory cytokines (such as IL-10, TGF-*β*), and involvement in tissue repair phase are considered as indicators of M2 differentiation. The different origin of the cells together with the functional plasticity of macrophages can explain the phenotypic and functional heterogeneity of KCs observed upon different triggers of liver pathology.

### 7.1. Kupffer Cells in NAFLD

KCs are known to be involved in the control of inflammatory responses in NAFLD. In the early stages of the disease, hepatic macrophages expand rapidly and secrete cytokines and chemokines such as IL-1*β*, TNF*α*, CCL2, and CCL5, contributing to a paracrine activation of protective or apoptotic signaling pathways in hepatocytes and the recruitment of other immune cells [68]. TLR4 plays a pivotal role in KCs activation. Dietary factors, such as fructose, may contribute to altered intestinal motility, bacterial overgrowth, and increased intestinal permeability which is also associated with NAFLD [67].

**Figure 1:**
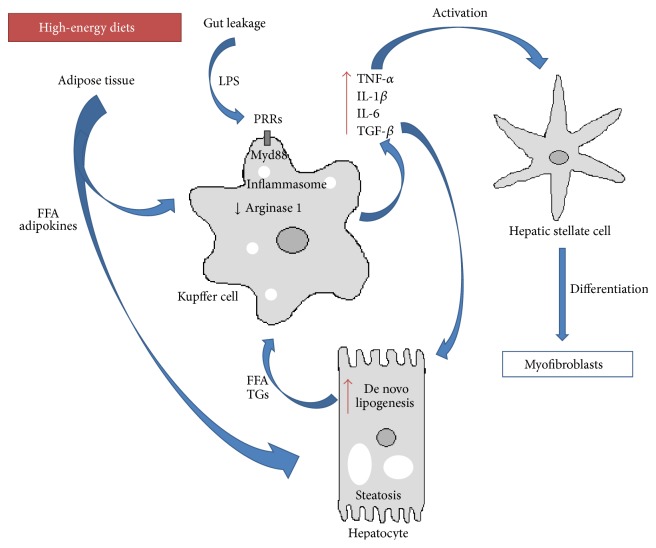
Kupffer cells and inflammation in NAFLD. Kupffer cells integrate a multitude of systemic and local stimuli, namely, inflammatory cues from diet-derived alterations in gut microbiota, as well as adipose tissue and hepatocytes signals of lipid metabolism impairments. Subsequent activation of Kupffer cells promotes a local inflammatory milieu that results in exacerbation of steatosis, hepatocyte dysfunction, and fibrogenesis. High-energy diets induce alterations in gut microbiome and increased permeability that result in augmented passage of pathogen-associated molecular patterns (PAMPs) such as lipopolysaccharides (LPS) to the portal vein. PAMPs interact with Kupffer cells through pattern recognition receptors (PRRs), activating innate immunity signal pathways (e.g., Myd88 or inflammasome) leading to the secretion of proinflammatory and profibrogenic cytokines. Increased adiposity and inflammation in adipose tissue caused by high-energy diets induce the release of free fatty acids (FFAs) and adipokines into circulation. FFAs and adipokines may on one hand contribute to Kupffer cell stimulation and on the other hand promote hepatic steatosis through enhanced FFA uptake and/or increase in* de novo* lipogenesis. Additionally, cytokine release by Kupffer cells sustain hepatic stellate cell differentiation into collagen producing myofibroblast and contribute to hepatocyte metabolic dysfunctions.

KCs are not only responsive to inflammatory signals but also to metabolic fluctuations (Figure 1). As referred previously, lipids* per se* and high-energy diets can be harmful to the liver. Evidences show that overload of lipids and cholesterol derivatives activate KCs in models of fatty liver disease and steatohepatitis [68]. A different study also showed that, under a high fat diet or upon FFA treatment, KCs are activated producing high levels of proinflammatory cytokines such as TNF*α* and IFN*γ* [69] (Figure 1). In this same study, increased levels of TLR4 were found in KCs. These results are in accordance with another study [70] where depletion of KCs protected against the development of high fat or high sucrose-induced steatosis. Furthermore, in a mouse model of steatohepatitis, mice with KCs derived from MyD88^−/−^ bone marrow donors had improved inflammation and steatosis phenotype [55]. Interestingly, KCs from mice exposed to high fat diet were shown to accumulate increased amount of free cholesterol and diacylglycerol.* In vitro* assays demonstrated that these fat-laden KCs were more responsive to LPS induced activation when compared to KCs from lean mice [68]. Accordingly,* in vitro* stimulation of mouse KCs with the SFA palmitic acid was shown to upregulate expression of TLR receptors [71]. Thus, FFA sensing by KCs may condition its responsiveness to proinflammatory triggers.

Insulin resistance is a predominant feature in NAFLD patients. Insulin signaling plays a critical role in modulating both glucose and lipid metabolism. Inflammatory mediators such as TNF*α* and IL-6 are highly associated with the development of insulin resistance in the setting of obesity. In mice, inactivation of NF-*κ*B mediated signaling, through specific deletion of ikk*β* in macrophages, was shown to impair the development of systemic insulin resistance under high fat diet. Nevertheless, adiposity was similar in macrophage ikk*β* deficient mice and wild-type controls under high fat diet [72]. Similar observations were obtained in bone marrow chimeric mice where JNK1 signaling was disrupted in hematopoietic cells [73].

### 7.2. Kupffer Cells and Hepatic Stellate Cells: The Path to Fibrosis

Liver fibrosis arises from dysregulation in the wound healing process elicited to dampen hepatocyte damage and is characterized by excessive matrix synthesis and altered matrix degradation. Fibrosis occurs as progressive liver accumulation of proteoglycans, glycoproteins, and collagens with predominance of types I and III fibrillar collagens and failure of physiological mechanisms of matrix turnover. Ultimately, the fibrotic process culminates in cirrhosis with distorted hepatic architecture associated with regenerating hepatocyte nodules surrounded by fibrotic septa and marked impairments in hepatic vascularization [74]. Development of hepatocellular carcinoma occurs in about one-third of individuals with cirrhosis [75]. Nevertheless, reversion of advanced fibrosis and even cirrhosis is possible and has been documented [74].

Myofibroblast that migrate and accumulate at sites of liver injury in response to autocrine and paracrine signals produced by neighboring cells constitute the cellular source of fibrosis during chronic liver diseases [74]. Although they are heterogeneous in composition a great part of myofibroblasts develop from liver resident HSC. HSCs are mesenchymal cells that comprise 5–8% of total liver cells. Their prime location is in the space of Disse between endothelial cells and hepatic epithelial cells. In quiescent state, HSCs store large amounts of Vitamin A in lipid droplets and can be identified by expression of desmin and glial fibrillary acidic protein (GFAP) [76]. Upon liver injury, HSCs sense hepatocyte damage and immune cell signaling and respond by transdifferentiation into active myofibroblast-like cells that express alpha-smooth muscle actin (*α*-SMA) and migrate to sites of injury. Besides secreting extracellular matrix proteins, HSCs secrete cytokines, growth factors that promote regeneration of hepatic epithelial cells, and angiogenic factors that modulate endothelial cell and hepatocyte proliferation. Prolonged activation of HSC causes fibrosis and during fibrosis regression the number of these cells is greatly reduced by induction of cellular senescence and apoptosis and return to quiescent state [76].

Genes regulating hepatocellular apoptosis and/or necrosis, genes regulating the inflammatory response to injury (TLR4, TNF*α*, IL-1*β*, and IL-6), genes mediating ROS generation, and genes coding for fibrogenic growth factors (TGF*β*1), vasoactive substances, and adipokines (Leptin) were shown to be critically involved in liver fibrogenesis [47, 77]. In particular TGF*β*1 polymorphism may confer susceptibility to NASH progression to fibrosis [77]. TGF*β* signaling in HSC stimulates activation, synthesis of extracellular matrix protein, and inhibition of its degradation [74]. KCs secrete TGF*β* and platelet derived growth factor (PDGF) that constitutes a potent mitogenic factor for HSC [47, 77] (Figure 1). Additionally, KCs through the secretion of IL-1 and TNF*α* lead to activation of NF-*κ*B signaling pathway in HSC promoting cell survival [78]. Interestingly, contact-independent coculture of KCs with HSC influenced gene expression in HSC turning the overall mRNA expression pattern more similar to what was observed in HSC isolated from* in vivo* mouse models of fibrosis. Among these genes were the NF-*κ*B-regulated genes* Il-6,* and tissue inhibitor of metalloproteinase 1 (timp1) [78]. Timp1 inhibits metalloproteinases (Mmps) that are able to cut collagen fibers and together modulate matrix degradation. Expression of both Timps and Mmps is tightly regulated according to activation of HSC in regular response to liver injury [74].

**Figure 2:**
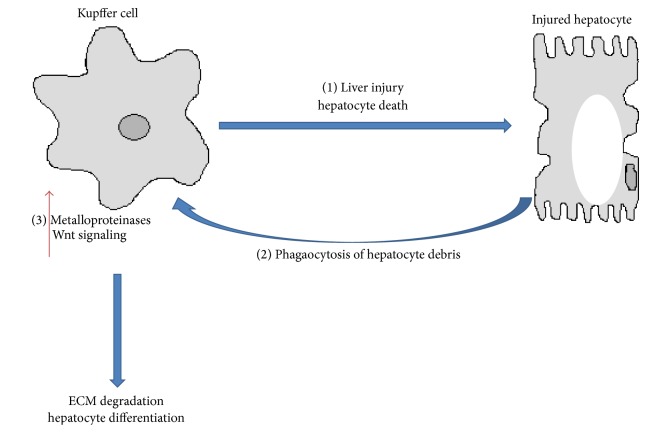
Role of Kupffer cells in fibrosis regression. Initial activation of Kupffer cells contributes to liver injury and hepatocyte cell death through the release of inflammatory cytokines (1). Phagocytosis of hepatocyte debris, however, may trigger an an antifibrogenic phenotype in Kupffer cells by inducing the expression of metalloproteinases that degrade collagen fibers (2). Phagocytosis may also increase Wnt signaling in KC and promote the differentiation of hepatic progenitor cells into new hepatocytes (3). A reduced phagocytic capacity of Kupffer cells may underlie impairments in antifibrogenic responses and contribute to the setting of fibrosis in NASH.

Macrophages can produce Mmps and contribute in a later stage of the wound healing process to fibrosis regression [79] (Figure 2). In a well described carbon tetrachloride model of reversible murine hepatic fibrosis, macrophage depletion during fibrosis acute phase leads to impaired activation of HSC and reduced scaring, while depletion during recovery phase reduced matrix degradation and fibrosis resolution [80]. In a similar mouse model of reversible fibrosis, monocytes were recruited into the liver where they were highly proinflammatory in initial stages, but upon stimuli of local microenvironment they differentiated into metalloproteinases producing macrophages necessary for fibrosis resolution [81]. Interestingly, phagocytosis of cellular debris was shown to trigger the phenotype switch, inducing expression of matrix degradation associated genes (Mmp9, Mmp12, and insulin growth factor 1) [81] (Figure 2).

Autophagy is a catabolic mechanism whereby unnecessary or dysfunctional cellular components are degraded in the lysosome. Autophagy-related (ATG) genes are necessary for the formation of the phagophore and autophagosomes, the initial steps in autophagy [82]. Autophagy in macrophages is critical for phagocytic functions. In a mouse model of fibrosis, inhibition of the autophagic gene Atg5 specifically in myeloid cells resulted in increased susceptibility to liver inflammation and liver injury, increased secretion of IL-1, enhanced monocyte recruitment, and increased hepatocyte apoptosis [82].

Phagocytosis of hepatocyte debris by liver macrophages/KCs was also shown to promote Wnt signaling upregulation. Wnt/*β*-catenin signalling is crucial for hepatic progenitor cell (HPC) differentiation and engagement into hepatocellular fate. Macrophage depletion during hepatocyte regeneration was shown to remove the stimuli for hepatocyte differentiation and HPC differentiated preferentially into cholangiocyte, forming biliary structures [83].

Macrophage plasticity may differ according to the nature of the stimuli leading to pathology. It is unknown whether impairments on macrophage phagocytosis and differentiation into matrix degrading prone-macrophages underlie fibrosis or hepatocellular differentiation triggered by NASH (Figure 2). Of note, impairment in KCs phagocytosis has been observed in high fat and high cholesterol diet-induced mouse models of NAFLD [84].

## 8. Conclusions

NAFLD is a rising concern that goes together with the increasing prevalence and incidence of obesity being a current epidemic problem. NAFLD pathogenesis is linked with insulin resistance, adipose tissue distribution, and dietary and genetic factors, which are risk factors not only for NASH but also for diabetes and associated pathologies.

From this review, it is clear that much remains to be understood regarding the mechanism of the disease. The lack of knowledge in relation to this pathogenesis becomes a hurdle in the path towards novel approaches for the prevention and treatment of the disease. Up to our days, prevention can only be based on calorie restriction and favorable dietary composition as well as exercise. More effective lifestyles/therapeutic methods should be addressed not only to prevent fat deposition, but primarily to avoid subclinical inflammation, where KCs play a prominent role. How the interdependent effects of diet microbiota inflammation directly impact liver dysmetabolism is an issue that remains to be elucidated. Nevertheless, from an interventional standpoint, this vicious cycle needs to be broken. If this hypothesis is proven to be correct, we should start to integrate the impact on intestinal microbiota composition and subsequent inflammatory responses in the nutritional value of foods, as a proxy for predicting the potential to evoke dysmetabolic states that are determinants of NAFLD development.
